# Automated Whole Animal Bio-Imaging Assay for Human Cancer Dissemination

**DOI:** 10.1371/journal.pone.0031281

**Published:** 2012-02-08

**Authors:** Veerander P. S. Ghotra, Shuning He, Hans de Bont, Wietske van der Ent, Herman P. Spaink, Bob van de Water, B. Ewa Snaar-Jagalska, Erik H. J. Danen

**Affiliations:** 1 Division of Toxicology, Leiden/Amsterdam Center for Drug Research, Leiden University, Leiden, the Netherlands; 2 Institute of Biology, Leiden University, Leiden, the Netherlands; Agency for Science, Technology and Research - Singapore Immunology Network, Singapore

## Abstract

A quantitative bio-imaging platform is developed for analysis of human cancer dissemination in a short-term vertebrate xenotransplantation assay. Six days after implantation of cancer cells in zebrafish embryos, automated imaging in 96 well plates coupled to image analysis algorithms quantifies spreading throughout the host. Findings in this model correlate with behavior in long-term rodent xenograft models for panels of poorly- versus highly malignant cell lines derived from breast, colorectal, and prostate cancer. In addition, cancer cells with scattered mesenchymal characteristics show higher dissemination capacity than cell types with epithelial appearance. Moreover, RNA interference establishes the metastasis-suppressor role for E-cadherin in this model. This automated quantitative whole animal bio-imaging assay can serve as a first-line *in vivo* screening step in the anti-cancer drug target discovery pipeline.

## Introduction

Traditional anti-cancer drug screens are performed using cell lines grown in 2D culture or using *in vitro* protein binding assays. Cancer progression, however, is a complex process of dynamic interactions between cancer cells and the organism that involves genetic alterations leading to deregulated survival and proliferation, angiogenesis, invasion, and metastasis [1]. Ideally, genes that play a role in this process are identified by *in vivo* ablation or silencing. Although genetic mouse models for cancer and human tumor cell xenotransplantation models in rodents remain essential, such systems are costly, slow, and less amenable to high-throughput assays for cancer drug target discovery. There is a clear need to develop fast, semi-automated *in vivo* systems for medium to high-throughput screening applications in preclinical target discovery and lead compound identification.

In this respect, zebrafish (ZF) offer a number of unique advantages for investigating the mechanisms that drive cancer formation and progression. ZF are vertebrates that can be raised in large numbers in a cost-effective manner. An almost complete genome sequence reveals that most cancer genes and tumor suppressor genes are highly conserved between ZF and humans (http://www.ncbi.nlm.nih.gov/genome/guide/zebrafish) and ZF form spontaneous tumors with similar histopathological and gene expression profiles as human tumors [2]–[4]. Importantly, xenotransplantation with human cancer cells is possible [5], [6]. ZF embryos that are used for this purpose lack an adaptive immune system, which increases the success of xenotransplantation while they provide a microenvironment where human tumor cells proliferate, migrate, form tumor masses, and stimulate angiogenesis [5]–[8].

ZF embryos are particularly useful for semi high-throughput microscopic analysis platforms as they are translucent, and can be maintained in 96 well plates. The optical transparency of ZF offers exciting research opportunities allowing visualization of the metastatic process at high resolution [8], [9]. Recent findings indicate that a wide range of pharmaceutically active compounds illicit physiological responses in ZF embryos and inhibit disease development similar to effects in mammalian systems [10]–[12]. These findings underscore the potential for a ZF embryo xenotransplantation model to be used in the anti-cancer drug discovery process. However, at this time, using ZF to screen for cancer relevant drug - and gene targets is limited by the lack of comprehensive automated bioassays. Here, we applied automated imaging and image analysis procedures to a ZF xenotransplantation model to develop the first semi-automated whole-organism quantitative bio-imaging assay for analyzing cancer dissemination in a vertebrate.

## Results

**Figure 1:**
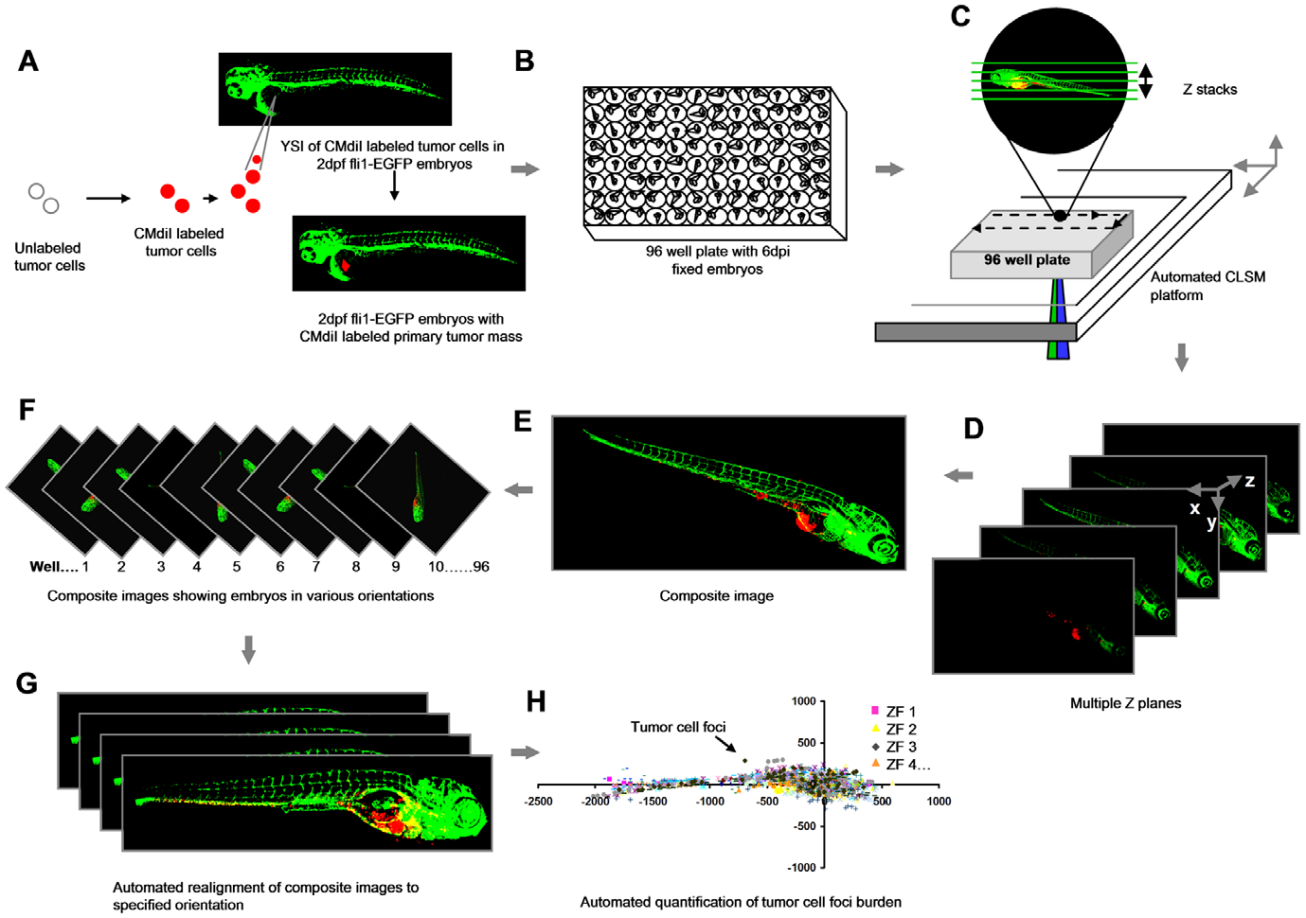
Schematic overview of the procedure. (**A**) Yolk sac implantation of CM-DiI labeled tumor cells into Tg (Fli:EGFP) ZF embryos 2 days post-fertilization. (**B**) Formaldehyde fixed 6 dpi embryos arrayed in 96 well plates. (**C**) Automated image acquisition using CLSM platform equipped with movable stage captures multiple Z stacks per embryo using 488 and 561 nm laser lines. (**D** and **E**) Automated creation of extended depth composite images. (**F**) Multiple extended depth images depicting embryos lying in different lateral orientations. (**G**) Automated uniform reorientation of images. (**H**) Scatter plot representing tumor foci burden in multiple embryos belonging to one experimental condition.

We developed a noninvasive, quantitative whole animal bioimaging method for dissemination of xenotransplanted human cancer cells in ZF embryos in 96-well format. All the steps are briefly outlined in **Figure 1**.

### Automated image capturing and pre-processing

CMDiI-labeled tumor cells were injected in the yolk sac of 2-day-old fli-EGFP embryos [13] and fixed 6 days post-implantation (dpi) (**Figure 1A**). Fixed embryos were arrayed in 96 well glass bottom plates for automated imaging (<5 minutes per plate) (**Figure 1B**). Epi-fluorescence microscopy failed to detect disseminated tumor cells due to excessive background from the primary tumor mass. Therefore, using a confocal laser-scanning microscope (CLSM) combined with an automated stage; multiple z stacks per embryo were captured for each well in a fully automated procedure (**Figure 1C, 1D** **and Video S1**). Confocal images were automatically converted into extended depth composite images (**Figure 1E**) that were rearranged to a uniform orientation (**Figure 1F and G**) to allow for automated quantitative image analysis (**Figure 1H**).

### Automated multiparametric analysis of cancer cell dissemination

**Figure 2:**
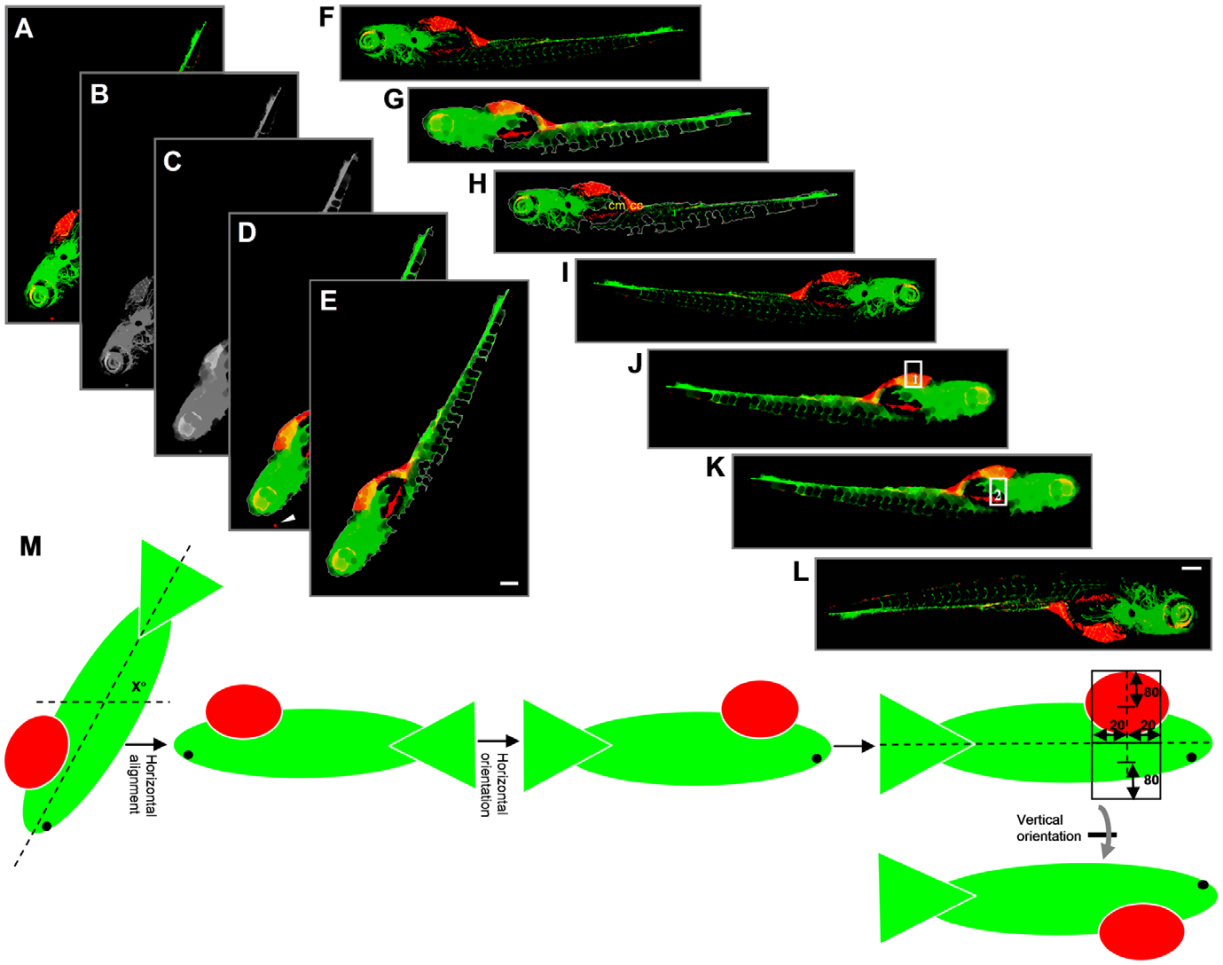
Outline of steps involved in embryo orientation. (**A**) Extended depth image of 6 dpi ZF embryo. (**B**) Grey value image from combination of green and red channels. (**C**) Blurred grey image after applying closing filter to optimize determination of outline. (**D**) Embryo segmented after applying intensity threshold and area filter. Arrowhead indicates a red object outside the outline that is excluded from segmentation. (**E**) Cropped image with only selected object. (**F**) Embryo rotated by x° for horizontal reorientation. (**G** and **H**) Determination of the x position value of the center of mass (*cm*) and center of centroid (*cc*). (**I**) Horizontal flip of the image only if *cm* is on the left side of *cc*, resulting in images with the head of the embryo always to the right side. (**J**) Image after applying closed filter to the combined green and red channel to get the outline of the embryo. Point lying at 75% distance from the extreme left of the embryo outline is calculated. Y-axis is drawn at this X-position from upper to lower outline. Upper rectangle 1 is drawn. (**K**) Lower rectangle 2 is drawn. (**L**) Vertical flip of the image only if red intensity in rectangle 1 is higher than in rectangle 2. (**M**) Schematic representation of calculations for steps **E–I**. Altogether, this procedure results in images where the head is on the right and the yolk sac is on the bottom of the image. Scale bar = 200 µm in **E** and **I**.

**Figure 3:**
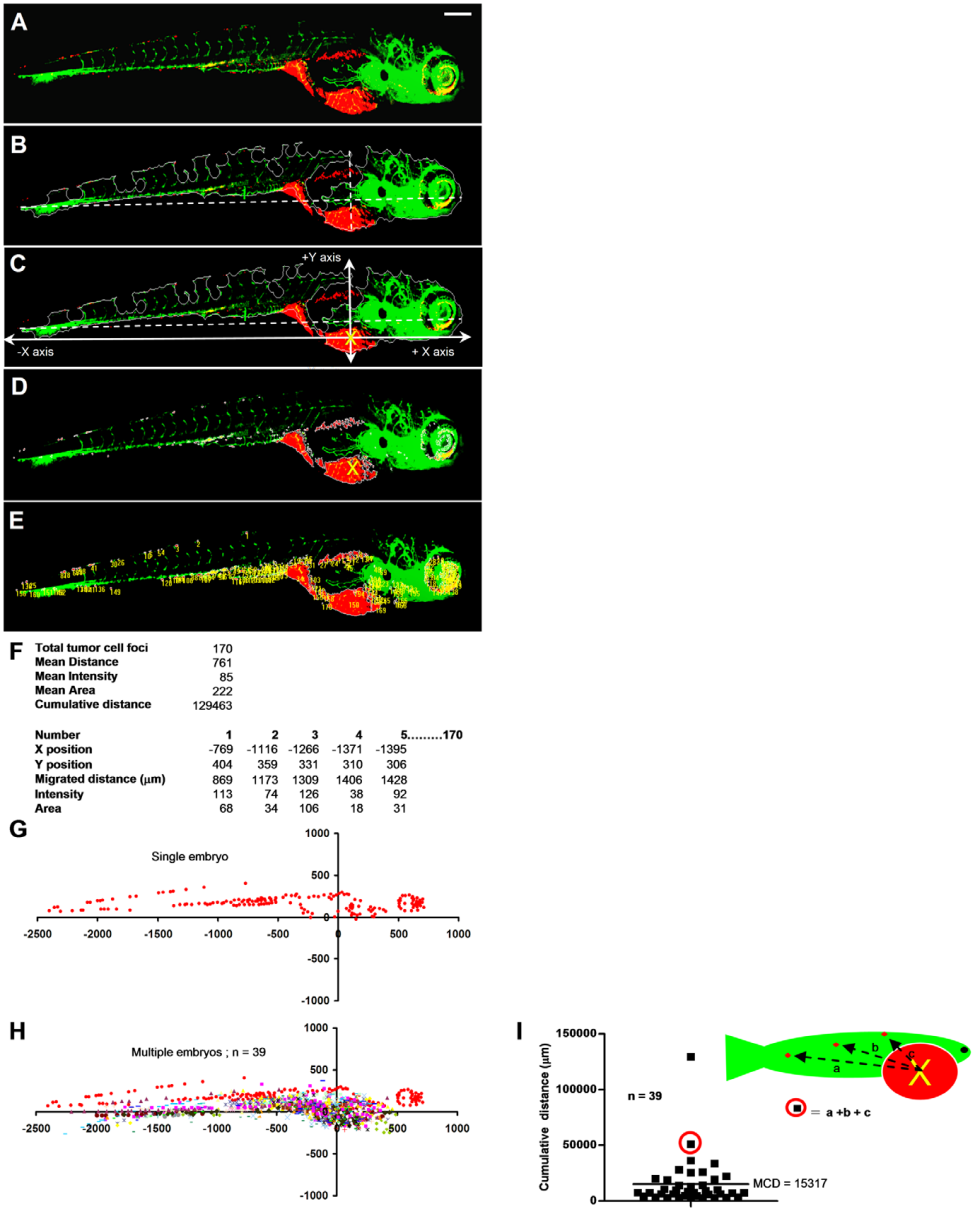
Automated multiparametric quantification of PC3 tumor foci. (**A**) Extended depth image of 6 dpi fixed embryo after realignment. (**B**) Embryo outline from segmented GFP channel and Y-axis intersecting X-axis at 75% from extreme left. (**C**) Calculated injection point at 75% distance from the extreme left and 75% from the top Y position. (**D**) Segmented red channel showing tumor foci burden in the embryo. (**E**) Identified tumor foci. (**F**) Multiple parameters of tumor foci burden calculated per embryo. Each number in the image corresponds to one tumor focus. (**G**) Tumor foci dissemination in a single embryo represented as scatter plot (coordinates 0,0 represents calculated injection site). (**H**) Combined scatter plot showing tumor foci dissemination from 39 injected embryos. (**I**) Quantification of cumulative distance (CD). Each filled square represents cumulative distance from injection point of all identified tumor foci in a single embryo. Mean cumulative distance (MCD) in the 39 injected embryos in this experiment is 15024 µm. Scale bar = 200 µm in **A**.

Having established conditions for automated imaging and image pre-processing of tumor cell implanted ZF embryos; we subsequently developed an algorithm for automated analysis of tumor foci burden in the post-processed ZF images. For this, Image-Pro based software was developed, which performed essentially three major functions (see materials and methods section for detailed information on the macro's). *1)* Reorientation of the images (**Figure 2**): all embryos were automatically reoriented to a horizontal orientation, with the head towards the right and the yolk sac towards the bottom. *2)* Determination of the injection position of labeled tumor cells (**Figure 3A–C**): the injection position was calculated from the images based on the segmented GFP channel (and confirmed by visual inspection using the red channel). *3)* Detection of tumor foci (**Figure 3D and E**): The red channel was segmented using an intensity threshold and minimum and maximum area filters.

Data were exported to excel and multiple numerical parameters were calculated to describe the tumor cell burden per embryo. These included total number of tumor foci, average distance of tumor foci from the injection site, and cumulative distance travelled from the injection site (**Figure 3F**). As only the cumulative distance parameter combined number of disseminated cells with their distance from the injection site, we reasoned that this parameter best reflected the tumor dissemination capacity (**Figure S1**). Data were represented as graphs displaying positions of tumor foci relative to the injection point (at coordinates x,y = 0,0) for each embryo (**Figure 3G**) or all embryos of a single experimental condition (**Figure 3H**). From these data, cumulative distance of all detected tumor foci was calculated per embryo (CD) and subsequently averaged for all injected embryos in one experimental group as a final quantitation of tumor cell dissemination (mean cumulative distance (MCD) (**Figure 3I** **and S1**).

**Figure 4:**
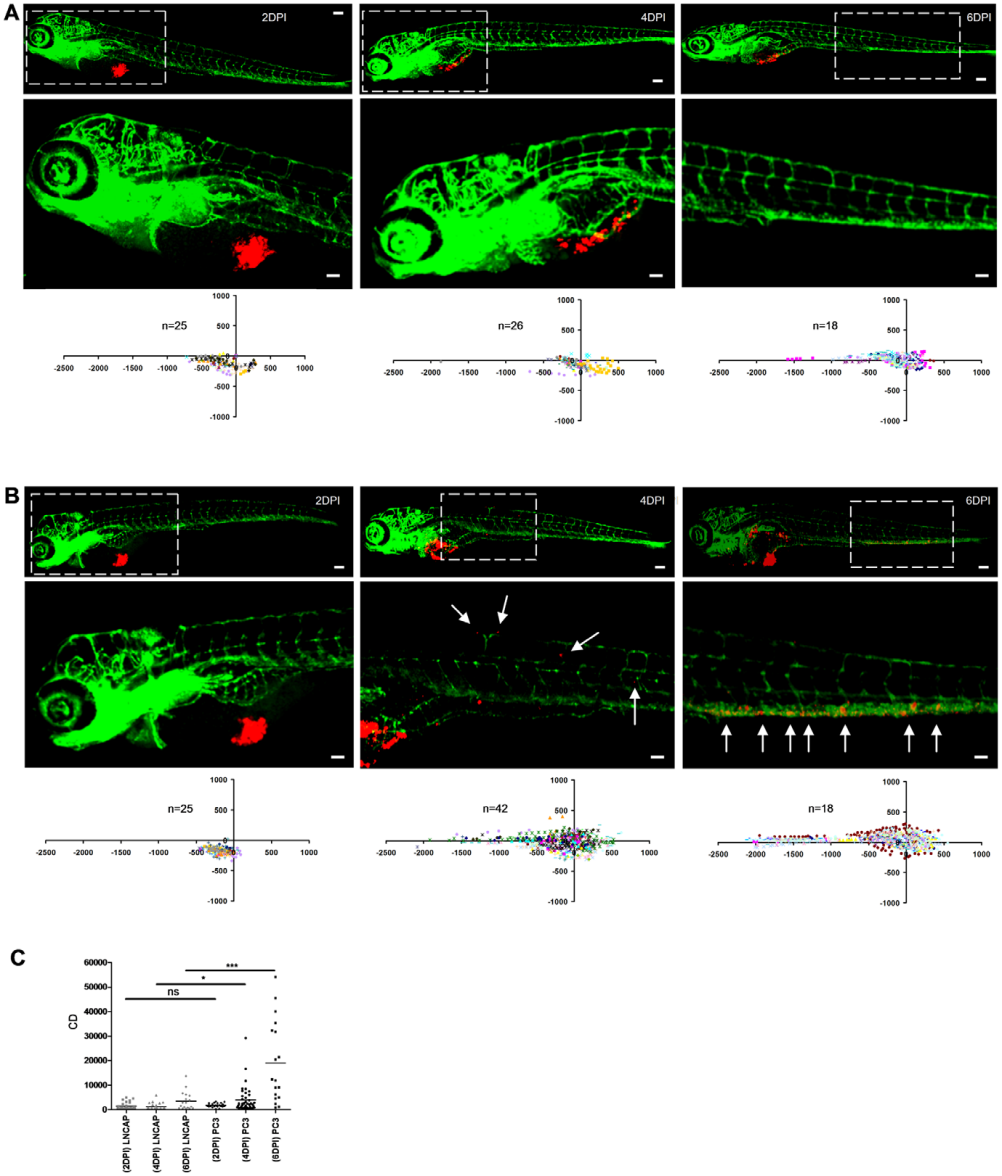
Determination of cancer cell dissemination kinetics. (**A** and **B**) LnCAP (**A**) or PC3 cells (**B**) were implanted and embryos were fixed at 2, 4, or 6 dpi for imaging (immunofluorescence images and automated image analysis (scatter plots)). Bottom row images (scale bar = 50 µm) show zoom-in of area marked by dotted line in top row images (scale bar = 100 µm). (**C**) CD at 2, 4 and 6 dpi for LnCAP (grey) and PC3-injected embryos (black) calculated from scatterplots in A and B, respectively. Statistical testing for difference between LnCAP and PC3 at different dpi is indicated. *p<0.05, ***p<0.001.

We performed experiments to determine the earliest time point that allowed robust discrimination between poorly aggressive and highly aggressive cell lines. Using LnCAP and PC3 as such an example for prostate cancer [14]–[19] we observed no difference at 2 dpi; MCD of PC3 could be distinguished from MCD of LnCAP at 4 dpi; and at 6 dpi MCD was markedly higher in PC3 compared to LnCAP with strong significance (**Figure 4**). Therefore, 6 dpi was chosen for analysis in all further experiments.

**Figure 5:**
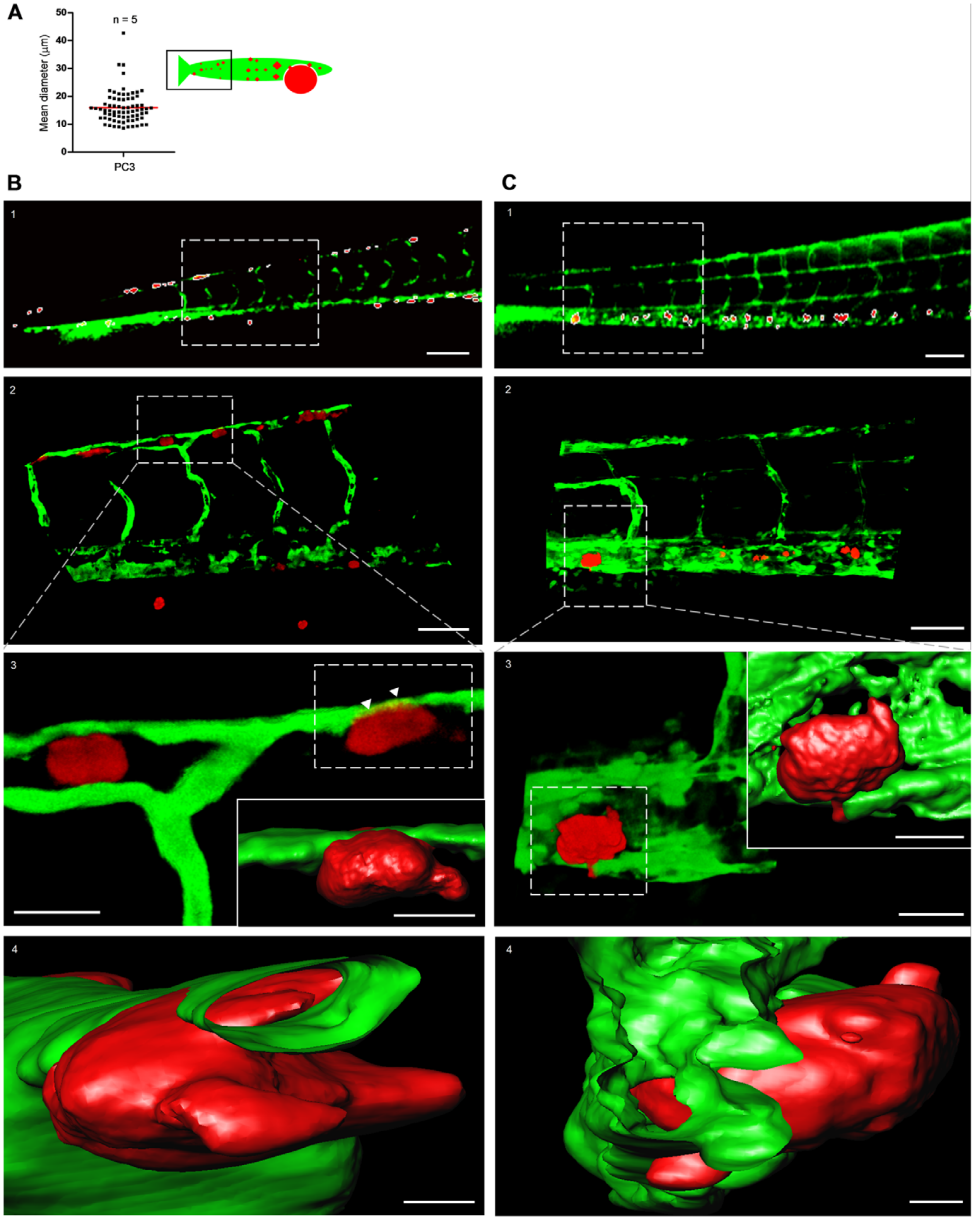
Characterization of tumor cell foci identified by macro using high-resolution imaging. (**A**) Quantification of the mean diameter of macro-identified tumor cell foci from the tail region of PC3-injected embryos. Data obtained from 5 embryos. (**B**) High resolution imaging of CM-DiI-labeled PC3 tumor cell foci. (**B1**) Macro-identified PC3 tumor cell foci. (**B2**) Zoom-in on area indicated in *B*1 shows tumor cells in association with host vasculature. (**B3** and **B4**) Three dimensional reconstruction and surface rendering of area in insert of **B2**; arrowheads point to tumor cell partly inside distal longitudinal anastomotic vessel (**Video S2** and **S3**). (**C**) High resolution imaging of PC3-mCherry tumor cell foci. **C1–4**, as **B1–4** for PC3-mCherry. Scale bar is 100 µm in **B1** and **C1**; 50 µm in **B2** and **C2**; 15 µm in **B3** and **C3**; 10 µm in insets in **B3** and **C3**; 5 µm in **B4** and **C4**.

**Figure 6:**
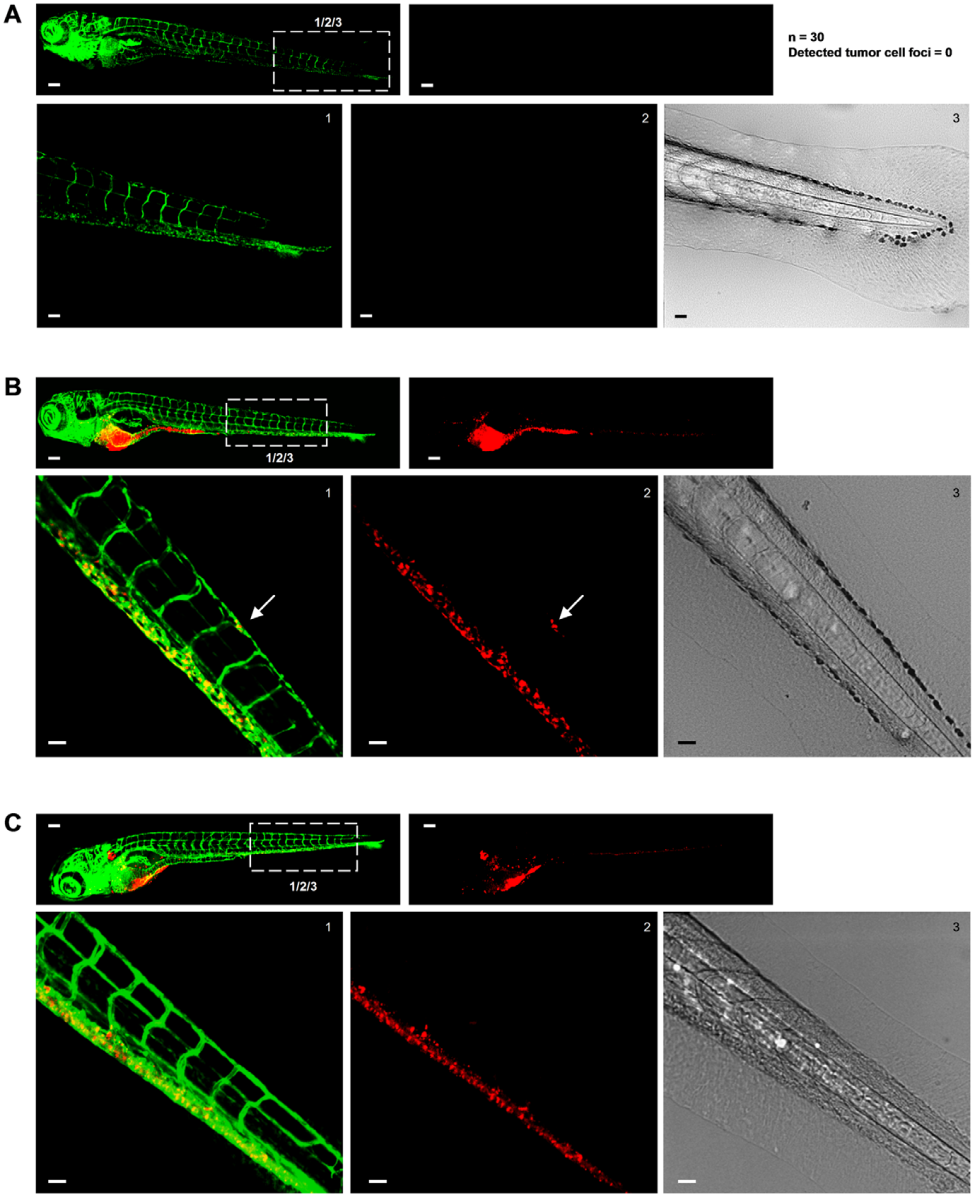
Excluding disturbance of the image analysis by autofluorescence signal from pigment cells. (**A–C**) In each case top left image shows green signal (Fli-EGFP) and top right image shows red signal for tumor cells. Bottom images show zooms of boxed area in top left image providing green (left) and red signal (middle) and transmitted light (right). Scale bar is 100 µm in images showing whole embryo and 50 µm in zoomed images. (**A**) Non-implanted fli-EGFP embryo imaged at 8 days post fertilization. Number of non-implanted embryos and number of tumor cells (falsely) detected by automated imaging and image analysis method is indicated at the right. (**B**) Fli-EGFP embryo implanted with CM-DiI-labeled PC3 imaged at 6 dpi. (**C**) Fli-EGFP Casper embryo implanted with CM-DiI-labeled PC3 imaged at 6 dpi.

To characterize tumor foci identified by this method, we calculated the mean diameter of segmented red objects in the tail region of PC3 implanted embryos. The average mean diameter was ∼15 µm with some larger objects up to ∼45 µm but no objects with average diameter <8 µm being selected for the analysis, fitting with the identification of individual tumor cells or small clusters (**Figure 5A**). We further analyzed identified tumor foci by high-resolution imaging, 3D reconstruction, and surface rendering (**Figure 5B** **and Video S2**). This confirmed and extended the finding that single tumor cells or small clusters were identified by the automated image analysis and showed tumor cells interacting with the host vasculature (**Figure 5B**). To rule out artifacts due to CMDiI labeling, we injected mCherry labeled PC3 cells. In complete agreement with the properties of red objects identified after injection of CMDiI-labeled PC3, individual PC3-mCherry cells were observed in close association with host blood vessels (**Figure 5C** **and Video S3**). Finally, experiments using unimplanted embryos (**Figure 6A**) and comparison of CM-DiI-labeled PC3 cells injected into standard fli-EGFP or Casper fli-EGFP embryos lacking all pigments (**Figure 6B and C**), ruled out any false positives due to autofluorescence of pigment cells.

Taken together, this method eliminates the need for visual scoring and enables automated generation and archiving of images and numerical data describing dissemination of tumor cells in a vertebrate organism. Moreover, for tumor cells identified by this automated bio-imaging assay, tumor-host interactions can be further studied in detail by high-resolution microscopy.

### Assay validation: correlation with behavior in mouse models and epithelial versus scattered phenotype

**Figure 7:**
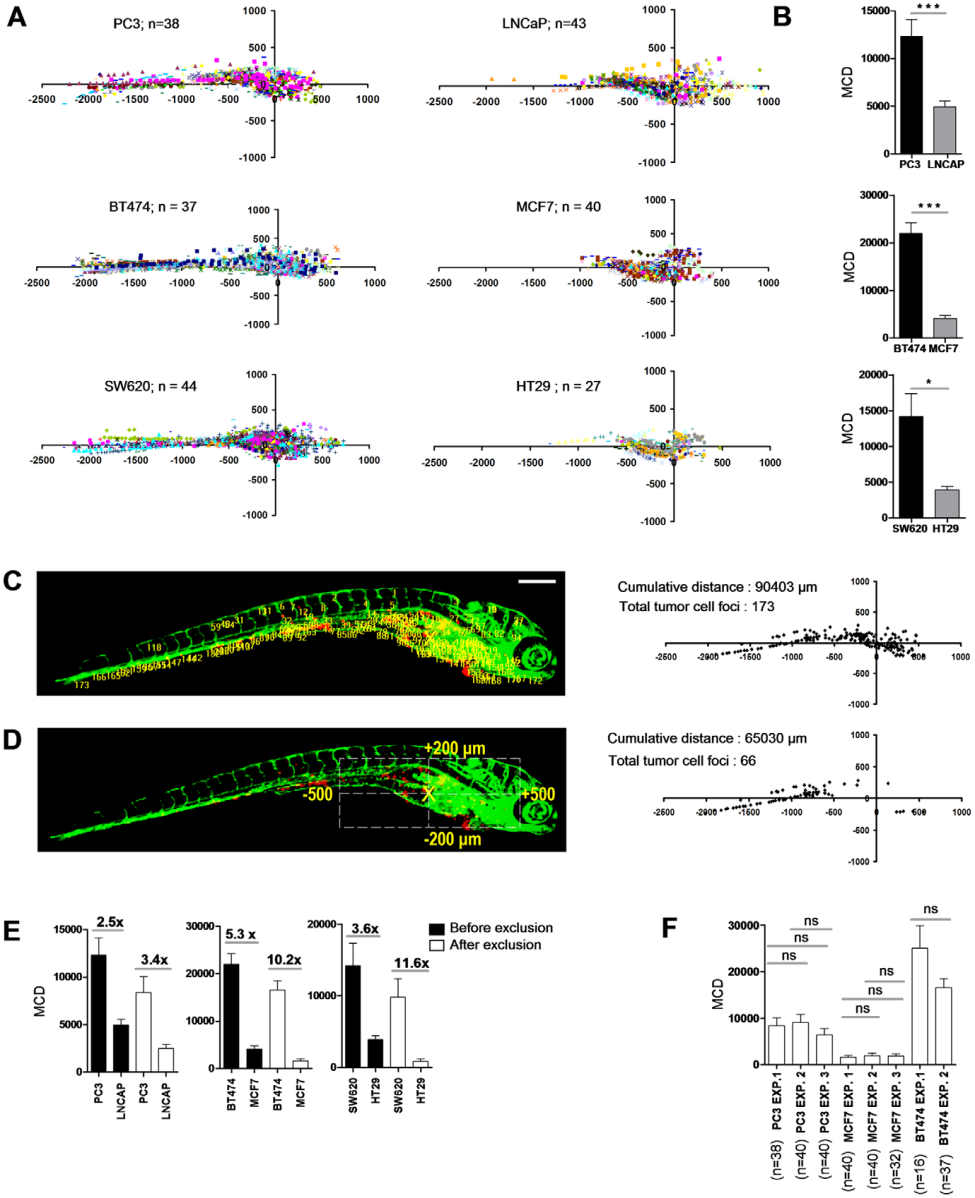
Differentiation between poorly and highly aggressive human cancer cell lines using automated bioimaging assay. (**A**) Scatter plot representation of tumor cell dissemination for indicated prostate (*upper* graphs), breast (*middle*), and colorectal cancer cell lines (*lower* graphs). Number of injected embryos from 2 biological replicates is indicated. (**B**) MCD determined from data represented in **A**. Data are presented as mean ± s.e.m. *p<0.05, ***p<0.001. (**C**) 6 dpi embryo injected with PC3 showing tumor foci burden determined from segmented red channel (*left*), and represented as scatter plot (*right*). (**D**) Automated determination of region for exclusion of tumor foci around implantation site and in area of intestinal development (*left*), and remaining tumor foci represented as scatter plot (*right*). (**E**) MCD before (*black*) and after exclusion (*white bars*) for the indicated prostate (*left*), breast (*middle*), and colorectal cancer lines (*right graph*). Fold difference between poorly and highly aggressive cell lines is indicated. Data are presented as mean ± s.e.m. *p<0.05, ***p<0.001. (**F**) MCD after exclusion for PC3 and MCF7 in multiple independent experiments demonstrates reproducibility. Data are presented as mean ± s.e.m.

To demonstrate the applicability of our automated platform to differentiate between poorly aggressive and highly aggressive cancer cells, three different panels of cell lines were analyzed: *1)* For prostate cancer, PC3 (highly metastatic in mouse models; prostate carcinoma/bone metastasis; androgen independent; scattered growth in 2D culture; mesenchymal markers) and LNCaP (very poorly metastatic in mouse models; prostate carcinoma/lymph node; expression of prostate differentiation markers; epithelial islands in 2D culture; epithelial markers) were analyzed [14]–[19]. *2)* For breast cancer, BT474 (metastatic in mouse models; breast invasive ductal carcinoma/duct; ER+/PR+/p53mutated; high Her2 expression; “weakly luminal epithelial like” phenotype in culture; reduced expression of epithelial markers) and MCF7 (very weak metastatic potential in the absence of ectopically expressed oncogenes; breast adenocarcinoma/pleural effusion; ER+/PR+/p53 normal; low Her2; epithelial islands in 2D culture; epithelial markers) were analyzed [20]–[23]. *3)* For colorectal cancer, SW620 (colorectal adenocarcinoma Dukes' type C/lymph node metastasis; scattered growth in 2D culture; mesenchymal markers) and HT29 (colorectal adenocarcinoma/colon; epithelial islands in 2D culture; epithelial markers) were analyzed [24]. Strikingly, for each cancer type tested, dissemination in this ZF xenograft assay significantly correlated with metastatic capacity reported in mouse models and/or characteristics known to be associated with cancer progression including differentiation markers or epithelial versus scattered phenotype (**Figure 7A and B** **; Figure S2**). These data validate this short-term automated bio-imaging method and show that it represents a powerful tool to predict aggressiveness of cancer cells in more complex, long-term *in vivo* systems.

Extensive ZF cellular movement takes place in the region of the yolk sac, where intestinal development occurs within the time frame of our analysis [25]. We wanted to exclude any influence of passive migration of implanted tumor cells due to this developmental process near the primary implantation area. For this reason, we expanded the macro with an additional step in which all tumor foci within a square encompassing this region were excluded from the analysis in an unbiased automated fashion (**Figure 7C and D**). Although this may lead to underestimation of the cumulative distance parameter, we found that this exclusion step even further widened the window between the non-aggressive versus highly aggressive cell types (**Figure 7E**). Moreover, analysis of several independent experiments using PC3, BT474, and MCF7, demonstrated that this methodology is highly reproducible (**Figure 7F**).

**Figure 8:**
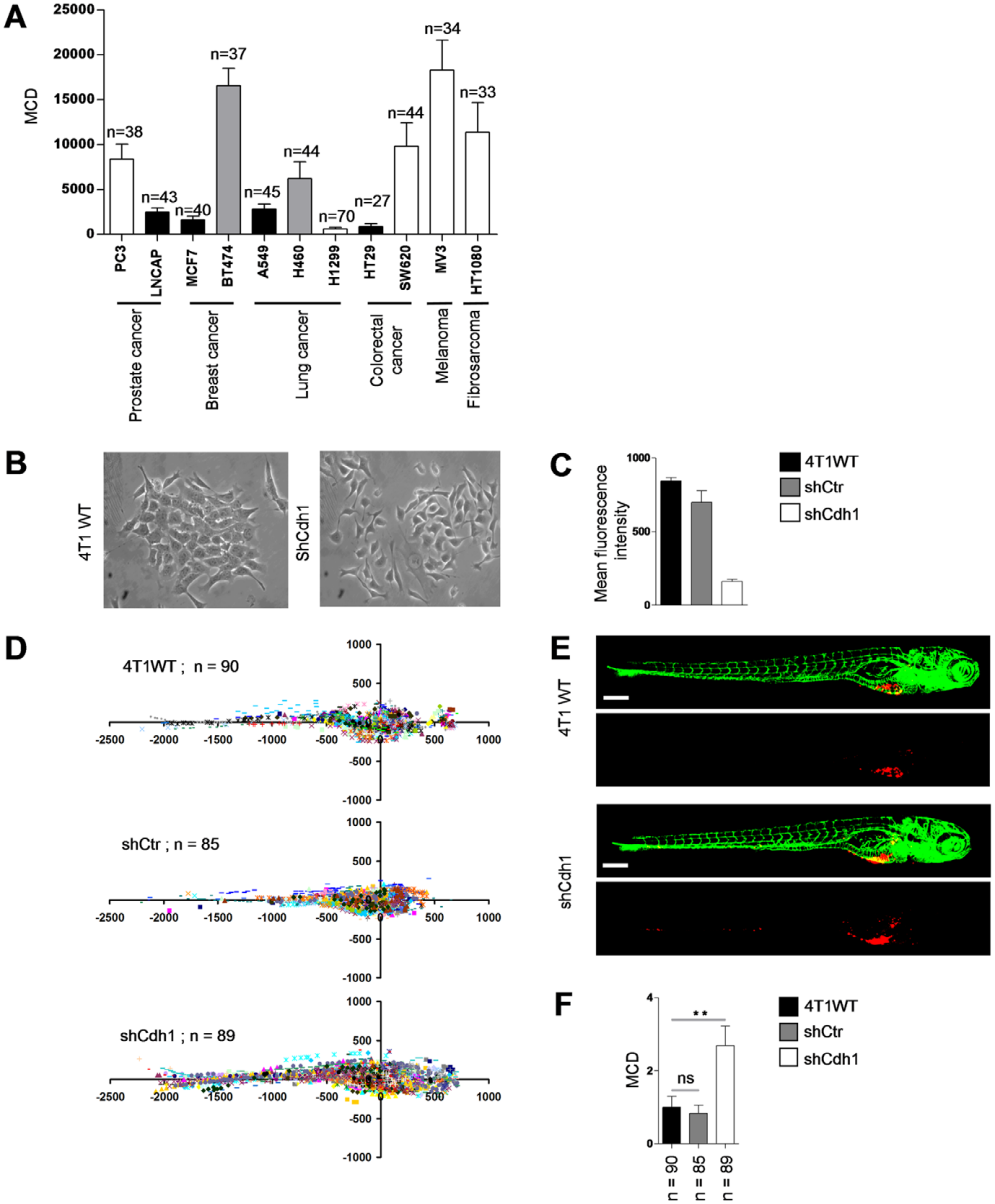
Differentiation between epithelial and mesenchymal cell types using the automated bioimaging assay. (**A**) MCD in a panel of human cancer cell lines from different origins. Number of injected embryos is indicated. *White bars* indicate cell lines showing a scattered phenotype in 2D cell culture. *Black bars* indicate cell lines growing as epithelial islands in 2D culture. *Grey bars* indicate cell lines with intermediate/mixed epithelial/mesenchymal characteristics. (**B**) 4T1 breast cancer cells growing as islands of loosely attached spindle-shaped cells (*left*) and completely scattered growth of 4T1 cells following E-cadherin silencing (*right*). (**C**) E-cadherin surface expression by FACS. (**D**) Scatter plot representation of indicated 4T1 variants. Number of injected embryos from 2 independent experiments is shown. (**E**) Representative images of embryos injected with indicated 4T1 variants. (**F**) MCD determined from data represented in **D**. Data are presented relative to wild type 4T1 as mean ± s.e.m. **p<0.01. Scale bar is 200 µm in **E**.

We expanded the analysis to a broader panel of human cancer cell lines from different origins including prostate, breast, lung, colorectal, skin, and connective tissue. Interestingly, when these lines were grouped according to their morphology in 2D culture and expression of epithelial or mesenchymal markers, there was a clear correlation of high dissemination with a scattered phenotype (one notable exception was HT1299, which did not disseminate effectively); all cell lines growing as epithelial islands had very low dissemination capacity (**Figure 8A** **; Table S1**). We functionally tested the role of one typical marker of the epithelial phenotype, the cell-cell adhesion receptor E-cadherin. For this, we used 4T1 mouse breast carcinoma cells that possess an intermediate phenotype, growing as clusters of loosely attached cells in 2D and expressing E-cadherin as well as some mesenchymal markers such as vimentin (**Figure 8B**). E-cadherin was silenced resulting in a fully scattered phenotype in 2D (**Figure 8B and C**) and these cells were injected in ZF to determine dissemination capacity. Indeed, shRNA targeting E-cadherin but not control shRNA strongly increased dissemination of 4T1 cells and the automated bio-imaging method allowed significant separation between 4T1shCdh1 on the one hand and 4T1Wt and 4T1shCtr on the other (**Figure 8D–F**).

Altogether, a short–term medium throughput fully automated whole-organism bio-imaging model has been developed that distinguishes with good reproducibility cancer cell types that grow as islands or have epithelial markers or have been shown to be poorly aggressive in mouse models from cell lines that are scattered or have more mesenchymal characterisitics or are known to metastasize in mice. It is compatible with RNAi for identification of regulators of cancer cell dissemination and allows switching from low-resolution fast imaging to high-resolution detailed analysis to study effects on tumor cell properties or tumor cell-host interactions.

## Discussion

Here, we developed a short term *in vivo* ZF xenograft assay that is compatible with automated imaging in 96 well plates and coupled to fully automated analysis of tumor cell dissemination. This assay represents the first automated whole organism bioimaging assay in a vertebrate that allows for studying aspects of cancer progression. Our findings show that this assay closely reflects the results obtained in more expensive and much lower throughput assays such as rodent xenografts.

The ZF xenograft model has been previously applied to cancer migration studies [5]–[9]. There are limitations to this model, including potential differences in the host microenvironment and the need to work at temperatures that are compatible with survival and migration of mammalian cells while at the same time being favorable for normal ZF physiology. Nevertheless, we have modified experimental conditions such that behavior of a large panel of human cancer cell lines from various origins closely resembles known behavior in rodent models. Moreover, our work provides this model with the automation in imaging and image analysis as well as with the statistical power required for application in screening procedures.

We provide proof-of-principle for such applicability by testing a known regulator of cancer cell migration. Using a panel of cell lines we show that this imaging platform can discriminate between cell types with more epithelial characteristics or growing in islands versus those with more mesenchymal characteristics or displaying a scattered phenotype). We then combine the assay with RNAi to silence the cell-cell adhesion molecule, E-cadherin. We rapidly obtain data from ∼90 animals per experimental group in two biological replicates supporting the inhibitory role of E-cadherin in cancer dissemination.

Taken together, this relatively fast medium throughput assay may be used as a first *in vivo* analysis platform in the target discovery pipeline. It provides the automation and statistical power to identify those hits from large-scale in vitro RNAi screening efforts that warrant more time- and money-consuming studies in mouse models. Future directions will include incorporation of similarly automated analysis of induction of tumor angiogenesis and tumor cell proliferation to capture multiple aspects of cancer progression within this bio-imaging assay.

## Materials and Methods

### Cell lines and ZF handling

All human cancer cell lines were obtained from ATCC and cultured according to the provided protocol. ZF and embryos were raised, staged and maintained according to standard procedures in compliance with the local animal welfare regulations. The transgenic ZF line Tg (fli1:EGFP) expressing EGFP in endothelial cells in wild type or “Casper” background, was maintained according to standard protocols (http://ZFIN.org). PC3-mCherry cells were generated using a pCMV-mCherry-bc-puro-Kl201 lentiviral vector (provided by Dr. R.C. Hoeben, Leiden University Medical Center, Leiden NL). 4T1-shCdh1 and 4T1-shCtr cells were generated by lentiviral transduction using TRC shRNA constructs (Sigma).

### Implantation procedure

Mammalian cells were labeled with lipophilic fluorescent cell tracker (CM-DiI; excitation maximum 553 nm, lot no. C7000; Invitrogen) according to the manufacturers instructions. The labeled cell suspension was loaded into borosilicate glass capillary needles (1 mm O.D.×0.78 mm I.D.; Harvard Apparatus) and the intrayolk injections were performed using a Pneumatic Pico pump and a manipulator (WPI). Dechorionated 2 days post-fertilization ZF embryos were anesthetized with 0.003% tricaine (Sigma) and positioned on a 10 cm petridish coated with 1% agarose. By controlling injection pressure and duration the number of injected cells was set at ∼100 per embryo as determined by standard cell counting of injection droplets. Injected embryos were maintained in egg water at 34°C and fixed 6 dpi with 4% paraformaldehyde.

### Automated microscopy and high-resolution imaging

Fixed embryos were manually arrayed into 96 well glass bottom plates (material no.655892; Greiner) with each well containing a single embryo. This was easily done in <5 min per plate. Image acquisition was performed by using a Nikon Eclipse Ti CLSM, which integrates advanced optics, fluorescence detection and scanning hardware in a single platform controlled by EZ C1 software. 488 and 561 nm laser lights were used to excite Tg (fli1: EGFP) embryos and DiI- positive tumor cells. Serial sagittal (lateral) sections were captured 6 dpi in an automated fashion (14×30 µm) using a Plan Apo 4X Nikon dry objective with 0.2 NA and 20 WD. For three dimensional images 0.5 µm step Z stacks (1024×1024 focal planes, 50–74 µm in depth) were acquired by using 40× Nikon dry plan fluor objective with 0.75 N.A and 0.66 WD.

### Image analysis software

Automated image pre-processing, automated analysis, and 3D reconstruction and surface rendering were performed by Image-Pro Plus-based software from Media Cybernetics.

### Extended depth of field

The software used Z-stack color images from the confocal microscope. The obtained gray images were pseudo colored with green for GFP and red for CM-DiI labeled tumor cells. A macro was built that uses both channels for batch processing of all images in a folder. First from the Z-stack a single, in-focus, composite image was made. Pixels in the Z-stack were analyzed. For every position, the pixel from the plane with the largest variance or local contrast was selected. This pixel was then used for the final composite image.

### Automated orientation of the embryos (Figure 2)

For the image analysis, all embryos should have the same orientation. For this, a macro was developed in which the images were modified so that all embryos were horizontally oriented, with the head towards the right and the yolk sac towards the bottom of the image.

Automated horizontal alignment: the color image was converted to a grey value image to give a combination of the GFP and red channels. The embryo was then segmented using mean intensity histogram value and minimum and maximum area filter. Then, a direction value of the segmented object was determined, which was used to give the embryo a horizontal position.

Automated horizontal orientation: the X position value of the center of mass was determined from the grey combined GFP and red channels. If this value was located left from the center of centroid, the image was flipped horizontally. In all cases, this provided images in which the head of the embryo was oriented to the right.

Automated vertical orientation: from the outer horizontal positions, the point lying on the X-axis at 75% distance from the extreme left of the embryo outline was determined. At this X-position the Y-axis was drawn from top to bottom outlines. A rectangle was drawn from −20 to +20 pixels horizontally from the calculated 75% point and vertically 80 pixels above the middle of the upper Y-axis. Another rectangle was drawn with identical horizontal parameters and vertically 80 pixels below the middle of the lower Y-axis. The average fluorescence intensity in the red channel was determined for both rectangles. If intensity was higher in the upper rectangle compared to the lower rectangle, the image was flipped vertically. The same procedure could be performed using the GFP channel in which case images were flipped if intensity was higher in the bottom rectangle. Visual inspection showed that this procedure, in all cases resulted in images where the yolk sac was oriented to the bottom of the image.

### Automated calculation of the injection position of the Cy3 labeled cells

First the leftmost and the rightmost X positions of the embryo outline were determined. From these X positions, a point lying at 75% from the extreme left was determined. Then, from this X position the uppermost and lowermost Y positions were determined. Subsequently, from these Y positions a point lying at 75% from the uppermost Y position was determined, which was designated as the arbitrary injection point. Visual inspection showed that the arbitrary injection point by this procedure, always resided within the primary tumor mass.

### Automated detection of CMDil labeled tumor cells

The pseudo colored red objects were segmented using a threshold value, and by applying a minimum and maximum area filter. After the segmentation, a mild watershed separation was applied. For all detected red objects the distance (in µm) from the injection point was calculated. A positive X value indicates tumor foci towards the head; a positive Y value indicates tumor foci towards the dorsal region of the embryo. Various other parameters, including number of objects, mean distance, cumulative distance, mean area, and mean intensity were calculated for segmented red objects.

### Processing of the results in excel

All data, together with the image were exported into excel. In excel 2 macro's were used: a) exclusion of tumor foci where [X absolute] is within 500 µm and [Y absolute] is within 200 µm from the calculated injection point; b) calculation of the average of all analyzed embryos. The major calculation chosen for representation of the data was “mean cumulative distance” (MCD) of tumor foci from injection position.

### Statistical analysis

Statistical analysis was performed with Prism 4 software (GraphPad) using two tailed, unpaired *t*-test. ns, not significant; *, P<0.05; **, P<0.01; ***, P<0.0001.

## Supporting Information

Figure S1 Automatically calculated cumulative distance (CD) in 6 dpi PC3 implanted embryos correlates with visual inspection of tumor cell dissemination. *A*, Scheme depicting concept of CD of tumor cell-foci. *B*, *left* images show CM-DiI-labeled tumor cells in red and GFP-endothelial cells of the Tg (Fli:GFP) line in green. *Right* images show only CM-DiI signal and calculated CD is indicated for each embryo.
(TIF)

Figure S2 Representative image of BT474 (*left*) and MCF7 (*right*) implanted 6 dpi embryo. Scale bar is 200 µm.
(TIF)

Table S1 Characteristics of cell lines used in Figure 8. Appearance in 2D culture and expression of epithelial versus mesenchymal markers is described for prostate cancer (black), breast cancer (red), lung cancer (green), colorectal cancer (blue), melanoma (orange), and fibrosarcoma (grey) cell lines.
(DOC)

Video S1 Combined multiple Z stacks of 6 dpi PC3-implanted embryo (range = 420 µm, step size = 30 µm, top = 180 µm, bottom = −210 µm). Red, CM-DiI-labeled tumor cells; green, GFP-endothelial cells of the Tg (Fli:GFP) line.
(AVI)

Video S2 Three-dimensional reconstruction and surface rendering shows tumor cell–vessel interaction for CM-DiI-labeled PC3 cell.
(AVI)

Video S3 Three-dimensional reconstruction and surface rendering shows tumor cell–vessel interaction for PC3-mCherry cell.
(AVI)
