# Supplementary figures and images for: Automated Whole Animal Bio-Imaging Assay for Human Cancer Dissemination
Source: PLoS One. 2012 Feb 8;7(2):e31281. doi: 10.1371/journal.pone.0031281 (PMC3275564; doi:10.1371/journal.pone.0031281)

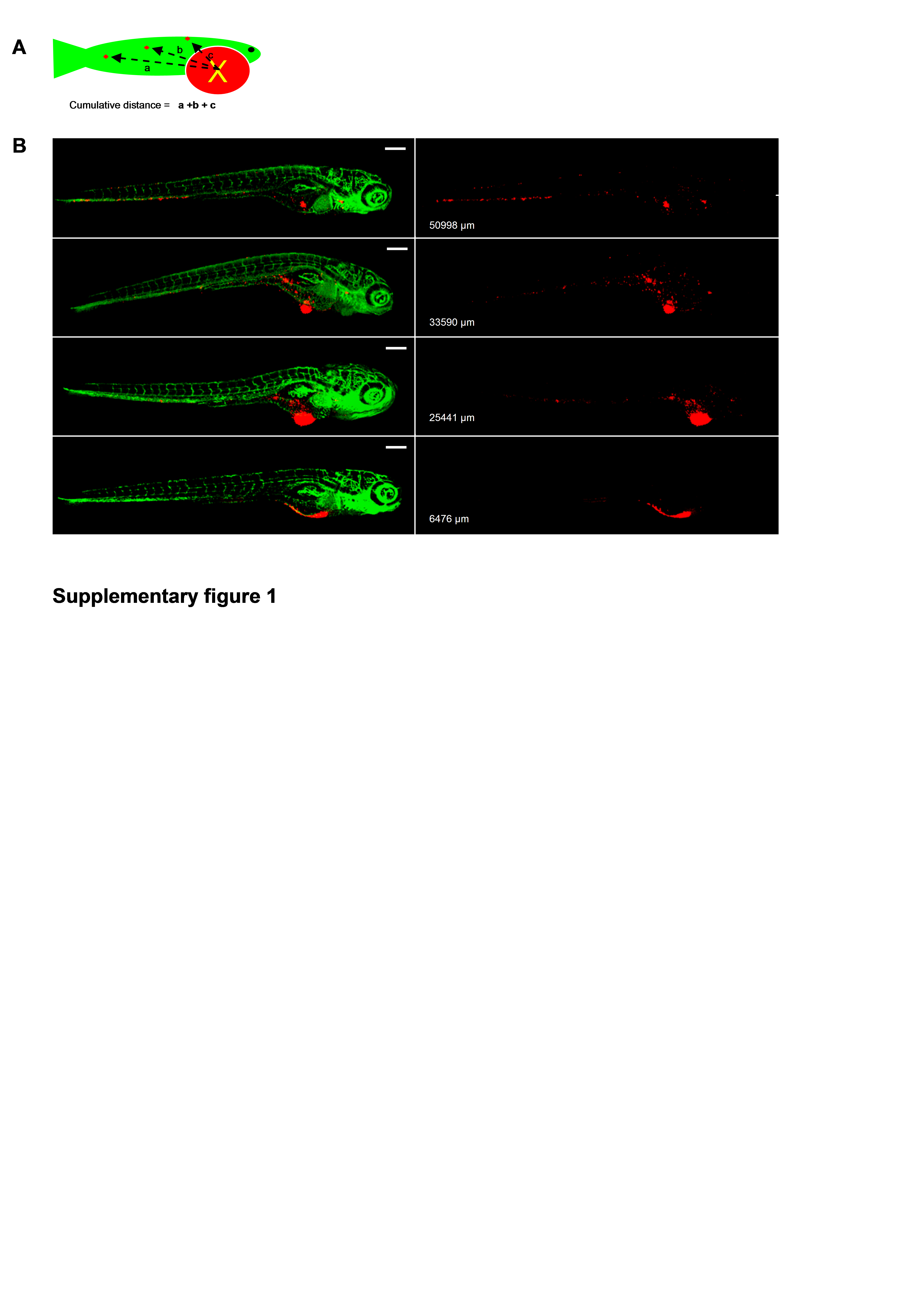

Supplement: Figure S1 — Automatically calculated cumulative distance (CD) in 6 dpi PC3 implanted embryos correlates with visual inspection of tumor cell dissemination. A, Scheme depicting concept of CD of tumor cell-foci. B, left images show CM-DiI-labeled tumor cells in red and GFP-endothelial cells of the Tg (Fli:GFP) line in green. Right images show only CM-DiI signal and calculated CD is indicated for each embryo. (TIF) [file pone.0031281.s001.tif]

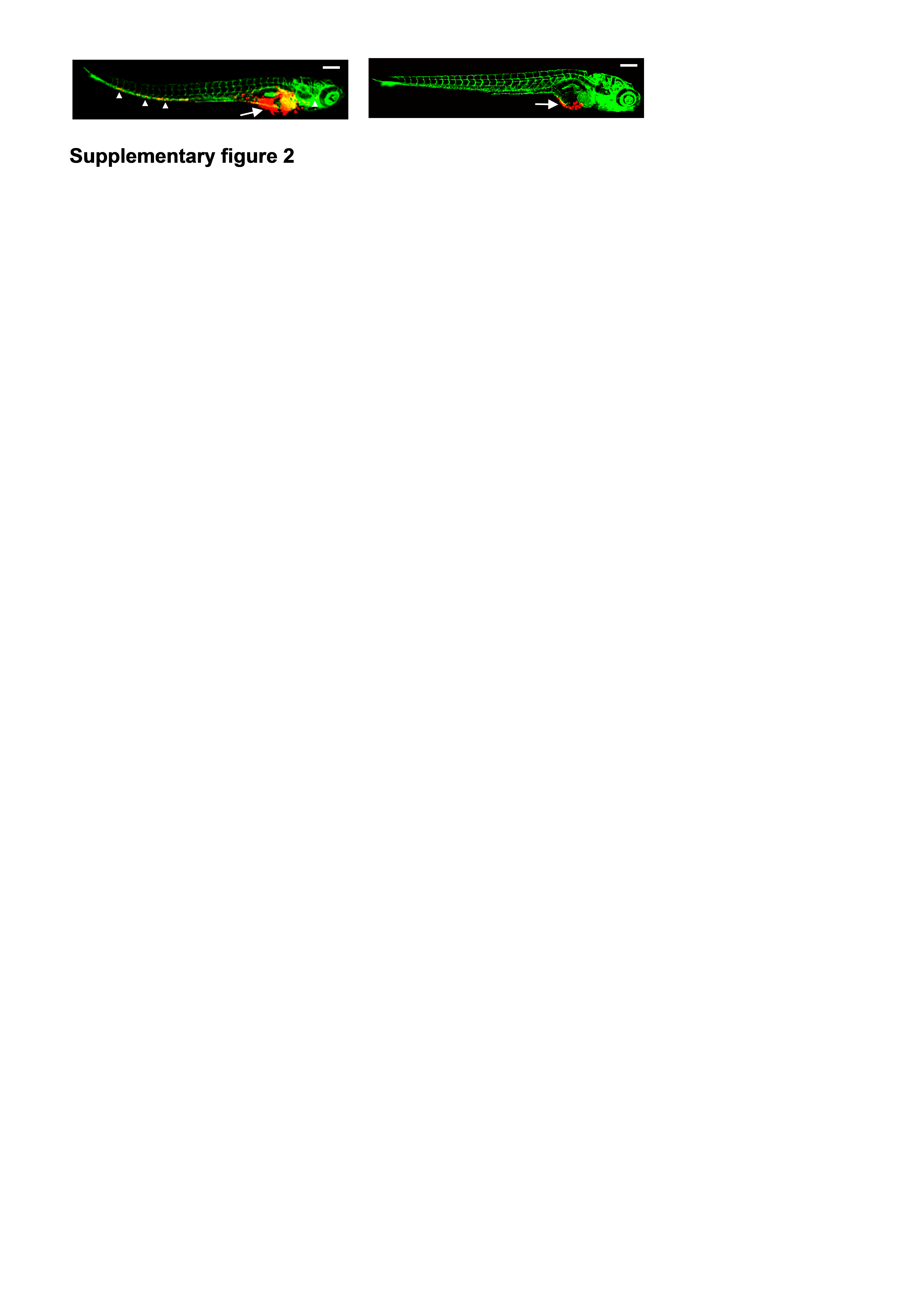

Supplement: Figure S2 — Representative image of BT474 (left) and MCF7 (right) implanted 6 dpi embryo. Scale bar is 200 µm. (TIF) [file pone.0031281.s002.tif]
