# Supplementary material for: Automated Whole Animal Bio-Imaging Assay for Human Cancer Dissemination
Source: PLoS One. 2012 Feb 8;7(2):e31281. doi: 10.1371/journal.pone.0031281 (PMC3275564; doi:10.1371/journal.pone.0031281)
Supplement: Table S1 — Characteristics of cell lines used in Figure 8. Appearance in 2D culture and expression of epithelial versus mesenchymal markers is described for prostate cancer (black), breast cancer (red), lung cancer (green), colorectal cancer (blue), melanoma (orange), and fibrosarcoma (grey) cell lines. (DOC) [file pone.0031281.s003.doc]

| **Cell line** | **2D morphology** | **Epithelial characteristics** | **Mesenchymal characteristics** |
| --- | --- | --- | --- |
| PC3 | Scattered |  | Deficient E-Cadherin dependent cell-cell adhesion due to absence of alpha catenin. Expression of vimentin, fibronectin, and N-cadherin (1,2) |
| LNCAP | Islands | Intact E-Cadherin dependent cell-cell adhesion, absence of N-Cadherin (3) |  |
| MCF-7 | Islands; interconnected colonies of polygonal cells | Markers typical of the luminal epithelial phenotype of breast cells: ER, E-cadherin, zonula occludens-1, and desmoplakin I/II (4) |  |
| BT474 | Mixed; clusters of loosely attached cells |  | Reduced extent of epithelioid markers (4) |
| A549 | Islands | Low E-cadherin (5) | Vimentin positive (5) |
| H460 | Mixed; clusters of loosely attached cells |  | Vimentin positive; E-cadherin negative (5) |
| H1299 | Scattered |  | Vimentin positive; E-cadherin negative (5) |
| HT29 | Islands | Intermediate snail expression, E-Cadherin positive, vimentin negative (6) |  |
| SW620 | Scattered |  | High snail expression, E-cadherin negative, vimentin positive (6) |
| MV3 | Scattered |  |  |
| HT1080 | Scattered |  |  |

Supplementary reference list:

1. Morton RA, Ewing CM, Nagafuchi A, Tsukita S, Isaacs WB (1993) Reduction of E-cadherin levels and deletion of the alpha-catenin gene in human prostate cancer cells. Cancer Res. 53:3585-90.

2. Härmä V, Virtanen J, Mäkelä R, Happonen A, Mpindi JP, et al. (2010) A comprehensive panel of three-dimensional models for studies of prostate cancer growth, invasion and drug responses. Plos one 5:e10431.

3. Tanaka H, Kono E, Tran CP, Miyazaki H, Yamashiro J, et al.(2010) Monoclonal antibody targeting of N-cadherin inhibits prostate cancer growth, metastasis and castration resistance. Nat Med. 16:1414-20.

4. Lacroix M, Leclercq G (2004) .Relevance of breast cancer cell lines as models for breast tumours: an update. Breast Cancer Res Treat. 83:249-89.

5. Gemmill RM, Roche J, Potiron VA, Nasarre P, Mitas M, et al. (2011) ZEB1-responsive genes in non-small cell lung cancer. Cancer Lett. 300(1):66-78.

6. Buck E, Eyzaguirre A, Barr S, Thompson S, Sennello R, et al.(2007) Loss of homotypic cell adhesion by epithelial-mesenchymal transition or mutation limits sensitivity to epidermal growth factor receptor inhibition. Mol Cancer Ther. 6:532-41.
